# Transient Ischemic Attack and Hypoventilation 12 Hours After Intra-vitreal Aflibercept Injection

**DOI:** 10.7759/cureus.13488

**Published:** 2021-02-22

**Authors:** Tariq Hamadneh, Mohammad Shehadeh, Anas Yasin, Mohammad Akkawi, MeiXia An, Hasan Yamin, Murad Azamtta

**Affiliations:** 1 Ophthalmology Department, The Third Affiliated Hospital of Southern Medical University, Guangzhou, CHN; 2 Ophthalmology, California Institute of Behavioral Neurosciences & Psychology, Fairfield, USA; 3 Department of Ophthalmology, Faculty of Medicine and Health Sciences, An-Najah National University Hospital, An-Najah National University, Nablus, PSE; 4 Department of Internal Medicine, Faculty of Medicine and Health Sciences, An-Najah National University Hospital, An-Najah National University, Nablus, PSE

**Keywords:** aflibercept, transient ischemic attack, hypoventilation, vegf inhibitors

## Abstract

Given the role the vascular endothelial growth factor (VEGF) plays in controlling and preserving the integrity of the vascular endothelium intravitreal administration of anti-VEGF agents may affect the risk of thromboembolic events. This is particularly noticeable in patients who are at risk for atherosclerosis. Here, we present one of the first case reports of transient ischemic attack (TIA) together with hypoventilation secondary to aflibercept injection.

A 63-year-old female suffered TIA together with hypoventilation about 12 hours after the third administration of intravitreal aflibercept, which is a VEGF inhibitor for diabetic macular edema (DME). Upon presentation, she was confused, had right-sided weakness and her respiratory rate was six breaths per minute, all of these resolved within the next 24 hours. The serum tests, cerebrospinal fluid (CSF) analysis, brain imaging, and carotid Doppler ultrasound were unremarkable. An ophthalmic exam revealed signs of bilateral proliferative diabetic retinopathy with left macular edema. Detailed reports of similar cases are lacking in the literature. Hypoventilation and thromboembolic could be possible side effects of aflibercept that necessitate more investigation.

## Introduction

Vascular endothelial growth factor (VEGF), also known as vasculotropin, is a dimeric, heparin-binding, polypeptide mitogen with four isoforms produced from alternative mRNA splicing [1]. In actively proliferating vascular tissue, the VEGF gene is widely expressed, and VEGF is considered a critical mediator of physiological and pathological angiogenesis [1]. It activates endothelial cells, promotes cell proliferation and migration, and increases vascular permeability [2]. Angiogenesis inhibitors, for example anti-VEGF inhibitors, have been classically used to treat neoplasia. Later, its use was expanded to target retinal angiogenesis related to several retinal pathologies such as age-related macular degeneration (AMD) and diabetic macular edema (DME) [3].

Three commonly used intravitreal VEGF inhibitors-aflibercept (Eylea®, Regeneron Pharmaceuticals), bevacizumab (Avastin®, Genentech), and ranibizumab (Lucentis®, Genentech)-are effective as angiogenesis inhibitors and relatively safe for the treatment of DME, but only aflibercept and ranibizumab are approved by the Food and Drug Administration (FDA) for this indication [1]. Bevacizumab, which is not approved by the FDA for any ocular indication, is widely used for off-label treatment of DME [4-5].

Considering the role that VEGF plays in the regulation of vascular endothelium and maintaining its integrity, anti-VEGF agents may have the potential risk of thromboembolic events, even with intravitreal administration. This is particularly evident in patients at risk of atherosclerosis [6-9].

Here, we report a case of TIA and hypoventilation developed within 12 hours of aflibercept administration for DME. Informed consent was obtained from the patient for this study.

## Case presentation

A 63-year-old female patient was admitted to An-Najah National University Hospital (NNUH) intensive care unit (ICU) for a decreased level of consciousness and hypoventilation. Her past medical history was significant for hypertension and type II diabetes mellitus complicated by proliferative retinopathy and nephropathy. The patient’s symtoms started one day before presentation when she had sudden onset right-sided weakness with aphasia and vomiting of normal gastric content with up rolling eyes and saliva drooling followed by a decreased level of consciousness and shortness of breath. By further questioning, her family denied a history of previous similar episodes. There was no history of fever, photophobia, neck stiffness, falling, or any psychosocial problems. She had not traveled or had any contact with sick people.

She received six bevacizumab (Avastin® 1.25 mg in 0.05 ml per injection) injections during the last two years period for optical coherence tomography (OCT) confirmed DME. Then, she was prescribed aflibercept (Eylea® 2 mg in 0.05 ml per injection) [5]. Two injections were given over the past four months with no complications. However, she suffered her current symptoms only 12 hours after the third injection of aflibercept.

On presentation, the patient was confused; her vital signs were significant for hypopnea, with a respiratory rate (RR) of six breaths per minute, and hypoxemia with a normal alveolar-arterial gradient (A-a gradient). Her physical exam showed mild weakness in the right side of the body; the rest of her neurological and systemic examination was unremarkable. The patient was initially diagnosed as having a transient ischemic attack with hypoventilation and further investigations to look for underlying pathology were ordered.

Routine chemistry was unremarkable except for 2.7 gm of proteinuria. Imaging studies including chest x-ray, brain computed tomography (CT), brain magnetic resonance imaging (MRI), brain magnetic resonance angiogram (MRA), echocardiogram, and carotid Doppler ultrasound were all within normal limits. CSF analysis was also normal with no signs of pleocytosis, xanthochromia, or red blood cells. An ophthalmic exam was performed and revealed bilateral proliferative diabetic retinopathy with left macular edema. Optic discs were within normal limits.

She received non-invasive ventilation in the ICU, and she improved over 24 hours with complete recovery of power and respiration.

## Discussion

Aflibercept is a recombinant fusion protein consisting of the extracellular domain of VEGF and the Fc region of human immunoglobulin G1 (IgG1) [9]. In contrast to bevacizumab and ranibizumab which bind only VEGF-A, aflibercept also binds to VEGF-B and the placental growth factor, which are also important angiogenic factors [3,10]. Aflibercept was approved by the FDA in 2011 for the treatment of AMD, and in 2014 for the treatment of DME [11].

In the context of current evidence of a reduction in circulating VEGF levels after intravitreal anti-VEGF injection, concerns regarding their safety and systemic adverse effects are understandable [12]. Historically, the intravenous use of the bevacizumab in the management of various cancers had been associated with higher rates of cerebrovascular events [13-14]. Given the fact of systemic absorption, the occurrence of these adverse effects after intravitreal use is not surprising. For instance, a 2-year data from the IVAN trial showed a statistically significant higher risk of cardiovascular events with bevacizumab than with ranibizumab [15]. Other studies linked the use of aflibercept with an increase in stroke incidence especially in those patients already with high baseline risk [16].

Generally, the reduction in plasma free-VEGF levels correlates with elevated levels of circulating anti-VEGF agents, with the reduction in free-VEGF levels greatest with aflibercept and least with ranibizumab [17]. This along with the wider spectrum of angiogenic factors inhibition may explain why aflibercept can be associated with higher cerebrovascular accidents. This issue is further amplified in the elderly, patients with diabetes, and those with recent stroke who may be at increased risk at the time of administration [12,16].

On a molecular level, binding of aflibercept to VEGF-A not only decreases VEGF concentration but also acts as a potent pro-inflammatory signal for increased expression of atherosclerosis-associated inflammatory mediators and cytokines which is in part mediated by Toll-like receptor 4 activation on the endothelial cell surface [18].

Although a limited number of case reports described thromboembolic event post aflibercept use, this is the first case reporting a possible association between aflibercept, TIA, and hypoventilation given the negative result of all investigations [19-20]. Moreover, apart from this potential link between aflibercept and TIA, a synergetic effect of aflibercept to the atherosclerotic consequences of age, diabetes mellitus and hypertension may have contributed to the occurrence of the ischemic event in our patient.

## Conclusions

VEGF inhibitors are commonly used as angiogenesis inhibitors for many ocular diseases. Because VEGF plays an important role in regulating and preserving the integrity of the endothelium, VEGF inhibitors can contribute to the development of thromboembolic events especially in patients who are at risk for atherosclerotic vascular diseases. Given the potential of systemic absorption after intravitreal VEGF inhibitors administration, systemic adverse effects of these agents are understandable. However, the exact association between VEGF inhibitors use and the risk of thromboembolism is still not clear and detailed case reports regarding this issue are lacking. We recommend further studies to investigate the systemic adverse effects of VEGF inhibitors as intraocular injections, especially the possibility of thromboembolic events.
